# Supplementary material for: Spexin2 Is a Novel Food Regulator in Gallus gallus
Source: Int J Mol Sci. 2023 Mar 2;24(5):4821. doi: 10.3390/ijms24054821 (PMC10003256; doi:10.3390/ijms24054821)
Supplement: Supplementary file 1 [file ijms-24-04821-s001.zip › ijms-2252779-supplementary.pdf]

Supplemental Table

Table S1 Primers used in this study

Gene	GenBank No.	Sense/antisense	Primer sequence (5'- to 3'-)	Size (bp)
		Primers for cloning the coding sequence of *cSPX2*		
SPX2	KF601213	Sense	GGACAGAAGTCGTGGGAGAAT	308
		Antisense	TCAACAGAGACATAGCTATAGG	
		Primers for 5'-RACE and 3'-RACE PCR		
SPX2-L1	KF601213	Antisense	CAAGCTTAACCTGGAGGACTTCAGTGC	
SPX2-L2		Antisense	CTATGGTTTTACCCTTTGGAGCACACCCG	
SPX2-U1		Sense	CCTTATAGTTGAAATCGGGTGTGCTCCA	
SPX2-U1		Sense	CAAGCTTAACCTGGAGGACTTCAGTGCA	
		Primers for quantitative real-time PCR assay		
SPX2	KF601213	Sense	AATCGGGTGTGCTCCAAAGG	90
		Antisense	TATCTCCTGCCATAGCGTCC	
NPY	NM_205473	Sense	AGCCCAGAGACACTGATCTCAG	184
		Antisense	TGCATGCACTGGGAATGACGCT	
AGRP	NM_001398243	Sense	GGAACCGCAGGCATTGTC	163
		Antisense	GTAGCAGAAGGCGTTGAAGAA	
CART	KC249966	Sense	CGTCCCGAGAGAAGGAGCTGATC	123
		Antisense	ACTGCTCTCCGGCGTCGCACAT	
PMCH	NM_001195795	Sense	AGAGGAGCGATGCCTTTGCT	310
		Antisense	CTCTTCCCAGCATACATCTGAG	
POMC	NM_001398117	Sense	CTGGGGCTGCTGCTGTGTCA	207
		Antisense	GAAATGGCTCATCACGTACT	
β-actin	NM_205518	Sense	GTCACCAACTGGGATGATAT	188
		Antisense	GCCTGGATGGCTACATACAT	
